# Differential Redox State and Iron Regulation in Chronic Obstructive Pulmonary Disease, Acute Respiratory Distress Syndrome and Coronavirus Disease 2019

**DOI:** 10.3390/antiox10091460

**Published:** 2021-09-14

**Authors:** Lorena Duca, Sara Ottolenghi, Silvia Coppola, Rocco Rinaldo, Michele Dei Cas, Federico Maria Rubino, Rita Paroni, Michele Samaja, Davide Alberto Chiumello, Irene Motta

**Affiliations:** 1 General Medicine Unit, Fondazione IRCCS Ca’ Granda Ospedale Maggiore Policlinico, 20122 Milan, Italy; lorena.duca@policlinico.mi.it (L.D.); irene.motta@unimi.it (I.M.); 2 Department of Health Sciences, Università degli Studi di Milano, 20142 Milan, Italy; michele.deicas@unimi.it (M.D.C.); federico.rubino@unimi.it (F.M.R.); rita.paroni@unimi.it (R.P.); michele.samaja@unimi.it (M.S.); davide.chiumello@unimi.it (D.A.C.); 3 Department of Anesthesia and Intensive Care, ASST Santi Paolo e Carlo, 20142 Milan, Italy; silvia_coppola@libero.it; 4 Respiratory Unit, ASST Santi Paolo e Carlo, 20142 Milan, Italy; rocco.rinaldo@unimi.it; 5 MAGI GROUP, San Felice del Benaco, 25010 Brescia, Italy; 6 Department of Clinical Sciences and Community Health, Università degli Studi di Milano, 20142 Milan, Italy

**Keywords:** acute hypoxia, chronic hypoxia, hepcidin, iron, erythropoiesis, antioxidant barrier, redox imbalance

## Abstract

In patients affected by Acute Respiratory Distress Syndrome (ARDS), Chronic Obstructive Pulmonary Disease (COPD) and Coronavirus Disease 2019 (COVID-19), unclear mechanisms negatively interfere with the hematopoietic response to hypoxia. Although stimulated by physiological hypoxia, pulmonary hypoxic patients usually develop anemia, which may ultimately complicate the outcome. To characterize this non-adaptive response, we dissected the interplay among the redox state, iron regulation, and inflammation in patients challenged by either acute (ARDS and COVID-19) or chronic (COPD) hypoxia. To this purpose, we evaluated a panel of redox state biomarkers that may integrate the routine iron metabolism assays to monitor the patients’ inflammatory and oxidative state. We measured redox and hematopoietic regulators in 20 ARDS patients, 20 ambulatory COPD patients, 9 COVID-19 ARDS-like patients, and 10 age-matched non-hypoxic healthy volunteers (controls). All the examined pathological conditions induced hypoxia, with ARDS and COVID-19 depressing the hematopoietic response without remarkable effects on erythropoietin. Free iron was higher than the controls in all patients, with higher levels of hepcidin and soluble transferrin receptor in ARDS and COVID-19. All markers of the redox state and antioxidant barrier were overexpressed in ARDS and COVID-19. However, glutathionyl hemoglobin, a candidate marker for the redox imbalance, was especially low in ARDS, despite depressed levels of glutathione being present in all patients. Although iron regulation was dysfunctional in all groups, the depressed antioxidant barrier in ARDS, and to a lesser extent in COVID-19, might induce greater inflammatory responses with consequent anemia.

## 1. Introduction

Hypoxia, or lack of oxygen (O_2_), is characterized by decreased O_2_ arterial partial pressure (PaO_2_) and, consequently, decreased O_2_ supply to tissues. By generating reactive O_2_ species (ROS), hypoxia upregulates several pro-inflammatory cytokines [1]. Physiological hypoxia is also a strong inducer of hematopoiesis: when exposed to high altitude, healthy subjects compensate hypoxia by augmenting red blood cell (RBC) production within a few days [2]. In this context, hypoxia adaptation implies increased need for iron to synthesize hemoglobin (Hb) to support hematopoiesis, which is met by augmenting iron entry in the circulation from duodenum, macrophages, and hepatocytes. By internalizing the iron transporter ferroportin, the protein hepcidin is known to down-regulate iron handling [3]. Hence, suppressing hepcidin synthesis through pathways that involve erythropoietin (EPO) release and iron redistribution [4], the hematopoietic compensatory response to hypoxia at altitude in healthy individuals is protected [5,6]. More specifically, iron handling is modulated by signals other than hematopoiesis, such as the size of systemic iron stores and the inflammatory response [7]. In fact, when in excess, free iron, or non-transferrin bound iron (NTBI), may act as a Fenton reagent in the Haber–Weiss reactions that result in increased ROS formation, with consequent unspecific cell damage.

In pathologic hypoxia, however, the system described above is subjected to relevant alterations. The main clinical situations in which hypoxia plays a key role are Chronic Obstructive Pulmonary Disease (COPD) and Acute Respiratory Distress Syndrome (ARDS). In addition, Coronavirus Disease 2019 (COVID-19) not only shares several features with ARDS, but it is often worsened in patients with pre-existing COPD [8]. In COPD, a lung disease characterized by persistent respiratory symptoms and airflow limitation, hypoxia and endothelial dysfunction contribute markedly to increased cardiovascular mortality and morbidity [9]. The physiological hematopoietic adaptation to hypoxia is observed only in a limited fraction of COPD patients: 40–50% of COPD patients instead fail to undergo hematopoietic adaptation and develop iron deficiency with anemia, a predictive risk factor for worse outcome [10,11]. Likewise, ARDS, a condition characterized by sudden onset of severe hypoxia without evidence of heart failure nor volume overload [12,13], is featured by high (34.9–46.1%) hospital mortality [14]. In ARDS, complications are mainly driven by ROS excess that upregulates the pro-inflammatory paths, thereby amplifying tissue damage and pulmonary oedema. In addition to COPD and ARDS, COVID-19 patients are often admitted to an Intensive Care Unit (ICU) because of severe hypoxia. Although COVID-19 may remember ARDS in its development and the severe respiratory presentation, new evidence supports the idea that COVID-19 is an entity separated from typical ARDS, as shown by different lung compliance and stronger endothelial inflammation [15]. The cytokine storm in ARDS and COVID-19, which anticipates the pro-oxidant and pro-inflammatory responses in hypoxia patients, consumes the endogenous antioxidant barrier, where coenzyme Q10 [16] and glutathione [17] play key roles. Inflammatory-driven iron toxicity may also correlate with an increased synthesis of sphingolipids [18] such as those derived from ceramide, which is cleaved by a ceramidase to produce sphingosine, which is phosphorylated to yield sphingosine-1-phosphate (S1P), a known mediator in COVID-19 syndrome [19].

The main objective of this study is to characterize the oxidative state in patients affected by either acute (ARDS and COVID-19) or chronic (COPD) hypoxia with the purpose to dissect the interplay among iron, inflammation and hypoxia, with two specific aims: (1) To evaluate a panel of redox balance biomarkers that may integrate the iron routine metabolism assays to monitor the patient’s inflammatory and oxidative state, to address their usefulness in validating interventions and to correct iron and redox imbalance. (2) To characterize the roles for hepcidin, NTBI and soluble transferrin receptor (sTfR) in the hematopoietic compensation to acute and chronic pathologic hypoxia.

## 2. Materials and Methods

### 2.1. Patients

The Milan Area 1 Ethics Committee approved the execution of the observational study (Prot.N. 5098/2020, 2019/ST/144, revised Prot.N. 0030016, 6 July 2021). We collected clinical data and blood samples from four groups of subjects/patients hospitalized at the San Paolo Hospital in Milan, Italy: (1) ICU patients diagnosed with ARDS; (2) ICU patients diagnosed with COVID-19; (3) Chronically hypoxemic stable COPD patients during routine evaluation; (4) age-matched healthy control subjects. ARDS and COVID-19 patients were recruited within 24 h after hospitalization, during which they were ventilated as necessary. Exclusion criteria were age < 18 y, chronic kidney disease and pre-existing hematopoietic disorders. The minimal sample size was established through a priori Power Analysis for attaining significant (*p* < 0.05, two-tailed) differences based on the expected changes of the most significant parameters (namely, Hb, hepcidin, RBC glutathione, glutathionyl Hb and NTBI, approximately +/− 30–50%) and the standard analytical reproducibility, assuming alpha = 0.05 and power = 0.80.

The definition of hypoxia was given from the following parameters: peripheral O_2_ saturation (SpO_2_) < 95%, and/or PaO_2_ < 75 mmHg in ambient air, and/or clinical need for a fraction of inspired O_2_ (FiO_2_) > 21% [20]. The arterial O_2_ content (CaO_2_) was calculated as:

CaO_2_ = (1.34 × [Hb] × SpO_2_) + (0.003 × PaO_2_) (1)

### 2.2. Blood Samples

After venous blood collection in both non-anticoagulated and EDTA vacutainers, samples were centrifuged for 15 min at 3000× *g*. Sera fractions were collected from non-anticoagulated vacutainers. Plasma and RBC concentrates were collected from EDTA vacutainers. All samples were stored at −80 °C for analysis.

### 2.3. Redox Imbalance

NTBI was assayed by high-performance liquid chromatography [21]. Hepcidin was measured by an EIA kit (Peninsula Laboratories, BMA Biomedicals, Augst, Switzerland). The dosage of sTfR was performed by an enzyme-linked immunosorbent assay (sTfR Human ELISA, Biovendor, Brno, Czech Republic). EPO was measured according to the manufacturer’s instructions by Human EPO/Erythropoietin ELISA Kit (Sigma-Aldrich, St. Louis, MO, USA). Malondialdehyde (MDA) was measured by BIOXYTECH LPO-586 Colorimetric Assay For Lipid Peroxidation (Oxis International, Burlingame, CA, USA), according to the manufacturers’ instructions.

The Ferric Reducing Antioxidant Power (FRAP) expresses the serum antioxidant capacity with respect to Fe(III)(2,4,6-tripyridyl-*s*-triazine)_2_Cl_3_ as oxidant. The reaction was carried out at pH = 3.6. A total of 5 µL of plasma was added to 300 µL of a freshly prepared FRAP solution [22] in a 96-well plate, and the absorbance was measured at 593 nm after 5 min of incubation at 37 °C against a blank. The standard curve was run using 6-hydroxy-2,5,7,8-tetramethylchroman-2-carboxylic acid (Trolox), a vitamin E soluble analogue as an antioxidant. Thus, 1 FRAP unit, defined as the reduction of 1 mol Fe^3+^ to Fe^2+^ is expressed as mmol equivalent of Trolox. All the reagents were acquired from Sigma Aldrich (St. Louis, MO, USA).

Soluble glutathione was assessed in the RBC fraction by the Glutathione Colorimetric Detection Kit (Invitrogen, Waltham, Massachusetts, USA). For deproteinization, 250 µL of RBCs was added to 1 mL of ice-cold 5% aqueous 5-sulfo-salicylic acid dihydrate (SSA, Sigma-Aldrich,) and incubated for 10 min at 4 °C. Samples were centrifuged at 2500× *g* for 10 min at 4 °C and the supernatant was collected for the measurement, according to the manufacturer’s instructions. The colorimetric reaction was read at 405 nm (Ensight™, Perkin-Elmer, Monza, Italy) and the measured absorbance referred to a standard curve.

### 2.4. Targeted Q10 and S1P Analysis

Serum (50 µL) was diluted with water (50 µL) and extracted with a methanol/ethanol (1:1, *v*/*v*) mixture (400 µL) [23]. The mixture was sonicated and extracted with an oscillator thermo-mixer (30 min, 5 °C, 1000 RPM). After centrifugation (10 min at 13,400 rpm), the protein debris was discharged and the clean supernatant was evaporated under a stream of nitrogen. The residues were dissolved in 50 µL of water and withdrawn in a vial.

The LC–MS/MS system consisted in a Shimadzu UPLC coupled with a Triple TOF 6600 Sciex (Concord, ON, CA, USA) equipped with Turbo Spray IonDrive (Concord, ON, USA). All samples were analyzed in positive mode with electrospray ionization. The instrument parameters were: CUR 35, GS1 40, GS2 40, capillary voltage 5.5 kV and source temperature 500 °C. Spectra were acquired by product ion scan of *m/z* 863.68 > 197.07 (coenzyme Q10) and *m*/*z* 380.254 > 264.26 (S1P). Declustering potential (DP) was fixed to 60 eV and collision energy (CE) was 30 ± 15 eV. Chromatographic separation was achieved on a reverse-phase Acquity HSS T3 column (1.7 μm, 2.1 × 100 mm; Waters, MA, USA) equipped with pre-column using water as mobile phase (A) and methanol as mobile phase (B), both containing 0.1% formic acid [24]. The flow rate was 0.4 mL/min, and the column temperature was 40 °C. The elution gradient (%B) was set as follows: 0–2.0 min (1%), 2.0–6.0 min (1–25%), 6.0–10.0 min (25–80%), 10.0–12.0 min (80–90%), 12.0–21.0 min (90–99%), 21.0–23.0 min (99–99%), 23.0–23.2 min (99–1%) and held until 30 min. Five microliters of clear aqueous supernatant was directly injected in LC–MS/MS. The chemicals (methanol, ethanol, formic acid) were purchased from Sigma-Aldrich (St. Louis, MO, USA). All aqueous solutions were prepared using purified water at a Milli-Q grade (Burlington, MA, USA).

### 2.5. Glutathionyl Hb

Glutathionyl Hb was measured in cold-water hemolysates of thawed RBC concentrates by Matrix-Assisted Laser-Desorption in a Time-of-Flight mass spectrometer (MALDI-ToF) [25]. All samples were run in quadruplicate. The Hb concentration of the diluted RBCs was measured at 420 nm (EnSight™, Perkin-Elmer, Monza, Italy) and compared to that of standard human Hb (Sigma-Aldrich, St. Louis, MO, USA).

According to their individual values (20–50 µmol/L), samples were diluted to a 10 µmol/L concentration. For the MALDI analysis, a 10 µL sample was mixed to an equal volume of freshly prepared sinapinic acid matrix (Sigma-Aldrich, MALDI-grade brand, 30 mg/mL in 50% *v*/*v* acetonitrile—0.1% trifluoroacetic acid). Four one-microliter aliquots were manually spotted in adjacent circular wells of a stainless-steel plate, air-dried at room temperature, and loaded into the Bruker Autoflex III mass spectrometer for measurement as described [26]. Peak areas were measured with a custom spreadsheet, and glutathionylated-to-free thiol HbSSG/HbSH was expressed as a percent ratio.

### 2.6. Statistics

Data are reported as mean ± SD. Data were tested for normality distribution using the D’Agostino–Pearson test. To assess differences among the groups, we used one-way ANOVA followed by the Tukey post-test if significant. The significance level was set at *p* = 0.05. All analyses were performed using GraphPad Prism.

## 3. Results

We recruited 49 patients and 10 age-matched healthy controls (Table 1). Among the COPD patients, eight were on continuous home O_2_ therapy (2 L/min O_2_ flow), but blood gas analysis was performed after >15 min breathing room air. Among the ARDS patients, 17 had pneumonia as the primary cause of respiratory disease, while 3 were hospitalized for gastrointestinal sepsis. Among the COVID-19 patients, six died within 28 days after diagnosis.

### Blood Gas Analysis, Hematopoietic Response and Inflammatory Response

**Figure 1:**
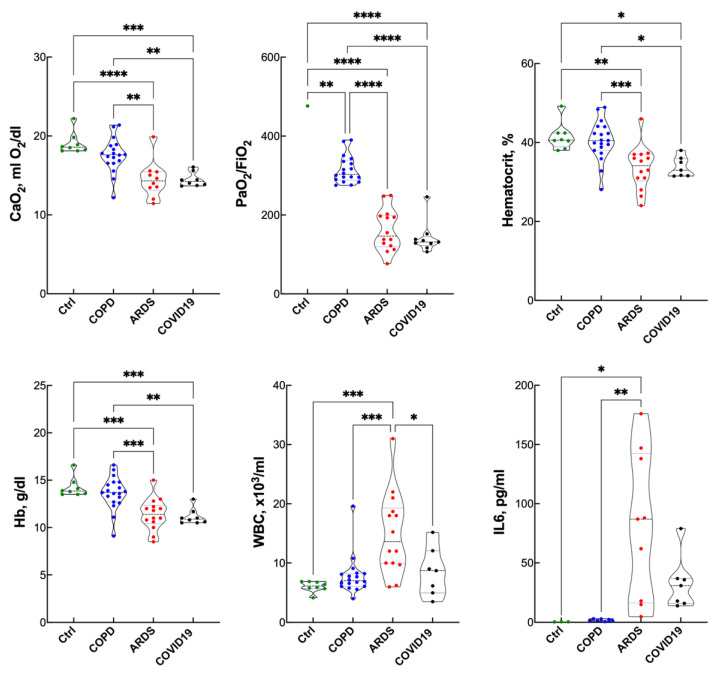
Hematopoietic, blood gas and inflammation data in controls, COPD, ARDS and COVID-19 (violin plots). Arterial blood gas analysis was not performed in Ctrl, and CaO_2_ in Ctrl was estimated assuming PaO_2_ = 100 mmHg. * *p* < 0.05; ** *p* < 0.01; *** *p* < 0.005, **** *p* < 0.0001 at the Tukey’s post-test.

Figure 1 shows arterial blood gasses, as well as hematopoiesis and inflammatory markers in the various groups. Blood gas analysis was not performed in the controls. No significant differences were detected for PaCO_2_ (43 ± 6, 46 ± 6 and 48 ± 10 mmHg), base excess (2.0 ± 3.5, 2.9 ± 3.2 and 1.0 ± 3.2 mmol/L), arterial pH (7.41 ± 0.04, 7.40 ± 0.06 and 7.40 ± 0.07), or plasma lactate (1.0 ± 0.4, 1.2 ± 0.3 and 1.0 ± 0.4 mmol/L) in COPD, ARDS and COVID-19, respectively. Although the PaO_2_/FiO_2_ ratio was higher in COPD than ARDS and COVID, all patients were within the criteria that define them as hypoxic.

The blood Hb concentration and hematocrit were lower in ARDS and COVID-19 compared to COPD and the controls. As expected, the inflammatory markers white blood cell (WBC) count and IL-6 were higher in ARDS and COVID-19 compared to control and COPD. However, IL-6 was surprisingly lower in COVID-19 compared to ARDS.

**Figure 2:**
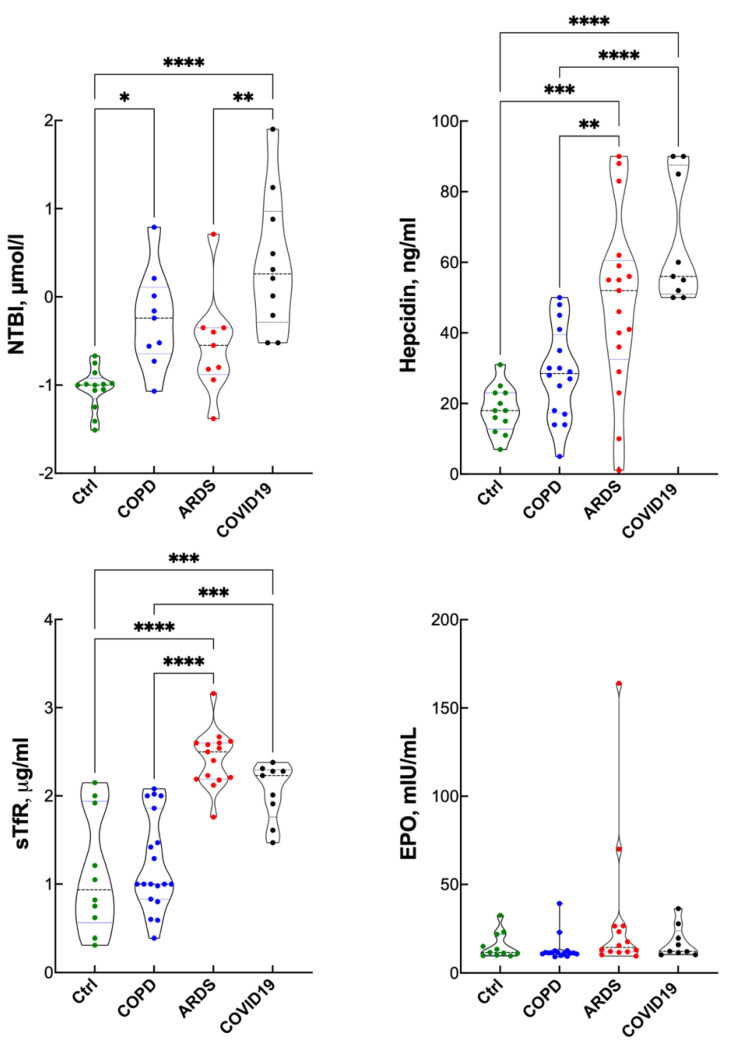
Iron and hematopoietic regulators in controls, COPD, ARDS and COVID-19 (violin plots). ANOVA for EPO was not significant. * *p* < 0.05; ** *p* < 0.01; *** *p* < 0.005; **** *p* < 0.0001 at the Tukey’s post-test.

Figure 2 shows some serum iron and hematopoietic regulators. NTBI, a toxic iron form, also a marker of the pro-oxidant challenge, was the highest in COVID-19 patients. Interestingly, NTBI was also increased in COPD patients when compared to the controls, while the difference ARDS vs. controls was borderline (*p* = 0.15). Hepcidin, an iron handling regulator induced by inflammation (average reference value 20 ng/mL [27]) was mostly increased in COVID-19 and ARDS compared to controls and COPD. Consistently with the lower Hb level, sTfR was higher in ARDS and COVID-19 than in COPD and the controls. With the remarkable exception of two ARDS patients, the EPO level was within the normal range, despite continuous hypoxic stimulus and decreased Hb.

**Figure 3:**
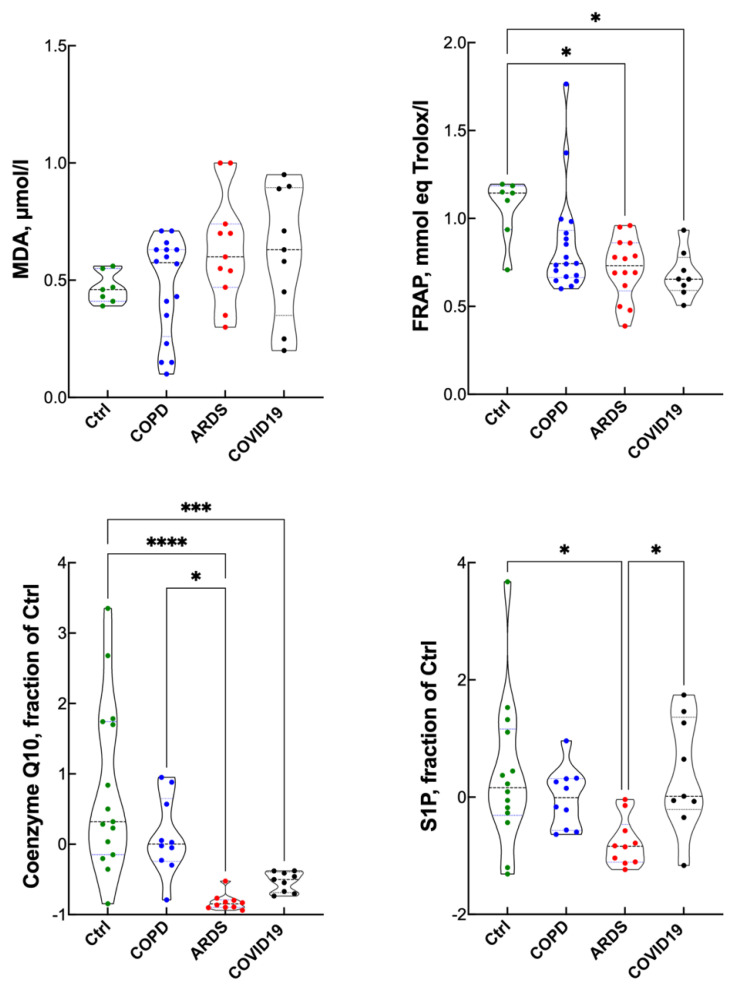
Redox imbalance parameters in controls, COPD, ARDS and COVID-19 (violin plots). ANOVA for MDA was not significant. * *p* < 0.05; *** *p* < 0.005; **** *p* < 0.0001 at the Tukey’s post-test.

Figure 3 shows some serum redox imbalance markers. MDA, a marker of lipid peroxidation and a sign of overt redox imbalance, was essentially the same in all the groups. FRAP, an index of the antioxidant barrier strength (reference values in normal subjects 1.5 mmol/L equivalent Trolox [22]) was reduced in ARDS and COVID-19 compared to the controls, with ARDS showing the lowest value. Coenzyme Q10, an important antioxidant and cofactor of the mitochondrial respiration, was depleted in all patients, especially ARDS. Because iron toxicity correlates with increased synthesis of sphingolipids, we measured S1P, which was slightly depleted in ARDS patients but essentially maintained in COPD and COVID-19.

**Figure 4:**
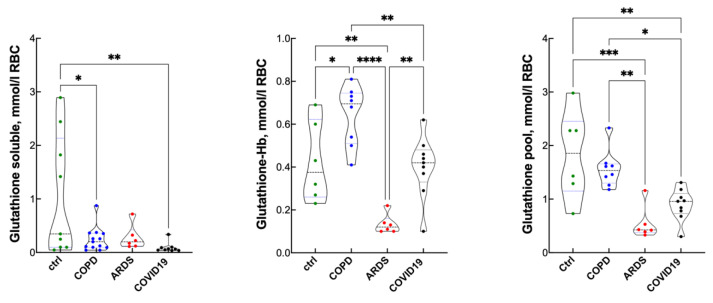
RBC soluble glutathione, glutathionyl-Hb and total RBC glutathione pool in controls, COPD, ARDS and COVID-19 (violin plots). * *p* < 0.05; ** *p* < 0.01; *** *p* < 0.005; **** *p* < 0.0001 at the Tukey’s post-test.

In the RBCs, glutathione occurs in two forms, either soluble (Figure 4, left panel) or bound to Hb as glutathione-Hb (Figure 4, middle panel). Glutathionyl Hb represents a minor form of Hb that indicates redox imbalance [26,28]. The right panel shows the total glutathione pool as the sum of the two forms. In COPD, the soluble glutathione form was less than the controls, but this was compensated by higher glutathione-Hb leading to an essentially conserved size of the total glutathione pool. By contrast, in ARDS and COVID-19, the pool was depressed, indicating an overall loss of glutathione units.

## 4. Discussion

In the present study, we characterize the redox state and iron regulators in pulmonary patients affected by COPD, ARDS and COVID-19. All these patients are classified as hypoxic. However, while ARDS and COVID-19 are relatively acute syndromes, COPD patients are instead a paradigm of relatively stable chronic hypoxic patients who may, nevertheless, be subjected to a certain degree of adaptation to hypoxia. All the hypoxic pulmonary patients studied here experienced greater iron pro-oxidant challenge as from higher NTBI. Remarkably, COPD patients are essentially successful in conserving their hematopoietic and iron regulator parameters, as well as a certain degree of resistance against redox imbalance from the maintained antioxidant barrier. By contrast, this ability is lost in acutely hypoxic patients who instead experience serious reductions in Hb and hematocrit, accompanied by uncompensated increases in hepcidin and sTfR, as well as depletion of the antioxidant barrier. As a whole, these observations might indicate that the systemic inflammation in ARDS and COVID-19 negatively interferes with the hematopoietic adaptation to hypoxia [19].

The loss of control of hepcidin release following inflammation and redox imbalance may indeed play a key role in the patterns observed here. Increased circulating hepcidin and sTfR, possibly secondary to increased pro-inflammatory C-reactive protein (CRP) and IL-6, might result in reduced iron release in the bloodstream, which triggers uncompensated anemia, much like in chronically diseased patients [29]. The inflammation in the blood vessel walls, together with dysregulated iron handling, plays a role in driving thrombosis formation, which is an important pathological factor in COVID-19 [30]. From a clinical perspective, increased hepcidin in ARDS might be associated with its antibacterial effect [31], a likely case when inflammation is a leading cause of bacterial infections. However, in viral infections such as COVID-19, such an increase is not justified in terms of hepcidin inhibitors [32] such as erythroferrone, which apparently does not play significant roles [33]. These data suggest that those inhibitors might be useful in preventing anemia onset in ARDS and possibly severe COVID-19, in which anemia represents an important comorbidity [34]. Remarkably, the increased hepcidin levels in ARDS may also be viewed in terms of evolutionary medicine as a natural body response to bacterial infection aimed at reducing pathogen proliferation through the limitation of iron availability [28].

To assess the role of iron regulation in pulmonary diseases, previous studies showed that hypoxic pulmonary vasoconstriction and the consequent hypoxic pulmonary hypertension are reduced by iron supplementation and exacerbated by iron deficiency [35,36]. In support of this, the present study focuses on a vicious cycle whereby increased NTBI that stems from COVID-19-related hyper-inflammation triggers further tissue damage that exacerbates inflammation [30,37]. As a matter of fact, lactoferrin and iron chelators have been recently proposed as a side therapy in COVID-19 to counteract the deleterious effects of NTBI [38]. Remarkably, COVID-19 patients display increased NTBI even compared to ARDS, a feature that, if further investigated in a wider sample, may discriminate COVID-19 from ARDS patients [38].

Redox imbalance is predicted to play a key role in the pathogenesis and development of ARDS and COVID-19 [39]. Although present data do not support overt oxidative damage (e.g., increased MDA), there are clear signs of consumption of the antioxidant barrier (e.g., FRAP test, Q10 and S1P) in acutely hypoxic patients. Antioxidant depletion may represent an early key feature in the patients’ deteriorating conditions, who here were studied within 24 h after hospitalization. Nevertheless, the perhaps not yet serious conditions of the patients before hospitalization are apparently already indicating anemia onset as well as clear signs of antioxidant depletion and inflammation [22,40].

Antioxidant depletion was here assessed through an array of markers. The coenzyme Q10, a marker of mitochondrial dysfunction, is particularly depressed in ARDS and COVID-19. As Q10 plays an important role in preventing lipid peroxidation [16], its administration may in principle help to reconstitute the antioxidant barrier in hypoxic patients. The importance of sphingolipids in the adaptation to inflammation and hypoxia-related redox imbalance is growing steadily [19]. In fact, healthy sojourners at altitude display elevated S1P [41], with S1P exerting protective effects on hypoxia-induced redox imbalance and proliferative as well as antiapoptotic effects on endothelial cells [42]. In RBCs, S1P acts by enhancing the glycolytic metabolic flux, which leads to the accumulation of 2,3-biphosphoglycerate that promotes O_2_ release by Hb [43]. In the context of this study, low S1P level in ARDS is a clear sign of lack of adaptation to hypoxia. The slow increase in S1P throughout the hospitalization in parallel to the decrease in inflammatory markers may make S1P a potential drug in hypoxic patients, as recently observed in animal models of hypoxia [44].

RBC glutathione may represent a further parameter that contributes to addressing the depletion of the antioxidant barrier in conjunction with FRAP, Q10 and S1P. Indeed, the level of soluble glutathione is lower in all classes of patients (borderline significance for ARDS) compared to controls and the normal reference value (1.4 ± 0.7 mM [45]). However, the glutathione units are in part bound to Hb to form glutathionyl Hb through a disulfide bond to the thiol group of the β93 cysteine residue. Due to its peculiar electrochemical redox potential, this minor form of Hb has been predicted to act as a redox buffer that scavenges oxidized glutathione in the oxidative phase and releases it in the recovery phase [46]. Remarkably, the level of glutathione bound to Hb is higher in COPD than it is in the controls, which re-establishes the size of the glutathione pool in chronically hypoxic patients. By contrast, the depleted glutathione pool in ARDS and COVID-19 indicates that, in acute pathological hypoxia, both glutathione functions, contributing to the antioxidant barrier as well as storage as glutathionyl Hb for later use, become depressed. This may address new therapeutical targets for the treatment of the redox imbalance in acutely hypoxic patients.

In the present study, it was not our purpose to investigate the time course of the response of acutely hypoxic patients to the onset of the complex pathophysiological situation associated with the diagnosis of ARDS and COVID-19. Thus, it is not surprising that, despite the increased need for hematopoiesis to optimize the blood O_2_ transport and to fight the redox imbalance, EPO remains within the normal range without being affected by the hypoxic conditions in ARDS and COPD patients, except for two patients. The lack of endogenous EPO response in the groups under study favors the pharmacological administration of EPO as an adjuvant to prevent anemia in ARDS and COVID-19, thereby delaying the need for blood transfusion [47]. EPO administration is indeed under investigation as a potential adjuvant treatment in COVID-19 [48]. Furthermore, the use of exogenous EPO in cases of iron overload, as in all the hypoxic groups under study, may help to reduce NTBI and prevent the well-known deleterious effects of iron in the Haber–Weiss reaction, thereby preventing damage to blood components and cells [49].

## 5. Conclusions

In this study, we show that hypoxia, a common feature in COPD, ARDS and COVID-19 patients, may translate in either redox imbalance or failed iron regulation in different ways depending on the clinical condition. All patients experience greater iron pro-oxidant challenge from increased NTBI, but COPD patients do not display substantial alterations in redox imbalance and failed iron regulation, perhaps due to stable chronic hypoxia that enables some form of adaptation. By contrast, ARDS and COVID-19 patients experience antioxidant barrier depletion (FRAP, Q10) and dysregulated iron handling (sTfR, hepcidin). The RBC glutathione pool reveals an additional novel biomarker of antioxidant resistance. The link between redox imbalance and failed iron handling due to the unregulated hepcidin may provide a mechanism in support of failed hematopoietic adaptation to pathological hypoxia.

## Figures and Tables

**Table 1:** Patients and subjects recruited for this study.

	Controls	COPD	ARDS	COVID-19
N (females)	10 (4)	20 (6)	20 (8)	9 (1)
Age, years	65 ± 9	74 ± 9	63 ± 19	67 ± 7

## Data Availability

Data is contained within the article.
